# The Black-White Mental Health Paradox Among Older Adults: Evidence From the Health and Retirement Study

**DOI:** 10.1093/geroni/igaa057.1935

**Published:** 2020-12-16

**Authors:** Lauren Brown

**Affiliations:** University of Michigan, Ann Arbor, Michigan, United States

## Abstract

Most studies of middle-aged adults find blacks have higher levels of psychological distress compared to whites but have lower risk of common psychiatric disorders. For instance, there is evidence of lower rates of depressive and anxiety disorders among blacks relative to whites despite large disparities in stress, discrimination and physical health in midlife—commonly referred to as the black-white mental health paradox. We examine evidence of the black-white paradox in anxiety and depressive symptoms among older adults. Data come from 6,019 adults ages 52+ from the 2006 Health and Retirement Study. Unadjusted models show older blacks report more anxiety and depressive symptoms than whites. After adjusting for socioeconomic factors, everyday discrimination, chronic conditions, and chronic stress, there are no black-white differences in anxiety and depressive symptoms. Findings suggest the black-white mental health paradox only extends into older adulthood for blacks living under similar stress and health landscapes as whites.

